# Supplementary material for: Chocolate intake and muscle pain sensation: A randomized experimental study
Source: PLoS One. 2023 May 24;18(5):e0284769. doi: 10.1371/journal.pone.0284769 (PMC10208501; doi:10.1371/journal.pone.0284769)
Supplement: S1 File — (DOCX) [file pone.0284769.s003.docx]

RESEARCH PROGRAM

Does dark chocolate influence experimentally induced masseter muscle pain in young healthy men?

- Or is it just chocolate preference?

**INTRODUCTION**

Chronic pain is a global health issue with massive costs for both the society and the individual (Goldberg et al 2011, Yokoyama et al 2007). A survey in Europe and Israel from 2003 showed that 20 % of the population has suffered from pain during more than 6 months. This can be interpreted as at least 20 % of the European population is suffering from chronic pain. This is in line with another survey, also reporting that around 20 % of the population has moderate to severe chronic pain. Already in 2005, the yearly total cost for pain was estimated to be €34 billion Euro. In the US, 100 million adults have reported to suffer from chronic pain. There, the yearly cost is estimated at 600 billion US dollars (Breivik et al 2006).

One of the most frequent loci for chronic pains is the orofacial region. Pain and temporomandibular disorders (TMD) show different prevalence depending on different study designs (Breivik et al 2006, Schiffman et al 2014). For example, one study claims that 5-12 % is affected by TMD (Schiffman et al 2014). Regarding the adult population specifically, one of these studies reported that about 7-11 % is suffering from TMD-pain. Further, epidemical surveys of TMD show that up to 5 % or 6 % of the population will have persistent pain severe enough to seek treatment (Svensson et al 2001).

Chronical pain is more common among women (Louca Jounger et al 2016, Dao and LeResche 2000). The pain can result in problems to chew and to open the mounth (Hallberg and Carlsson 2000, Thomas 2000). A recently published epidemiological study showed that 5.2 % of the women had orofacial pain at least once every week (Lovgren 2010). The mechanisms behind pain and behind the gender differences are still not yet fully known. There are also gaps in the knowledge regarding the causal associations behind pain. When the knowledge is increased regarding pain mechanisms and causes, new opportunities and treatment approaches will appear. One way to investigate pathophysiological mechanisms is the use of experimental pain models (Svensson et al 2001). The strength of experimental pain models is that it is possible to have groups of individuals that are healthy and in similar ages, sex, psychosocial statuses which in turn would decrease the surrounding factors that might affect the outcome, i.e. minimize the bias (Panucci et al 2010).

It is known already that different neurotransmittor agents, like serotontin can effect the well being and that it also is involved in pain reduction (Lesurtel et al 2008). Intake of carbohydrates can increase the serotonin production (Silva et al 2010) Cocoa contains a high amount of serotonin which has a positive impact of the well being (Nehlig 2013). It has been shown that a cocoa-enriched diet inhibits neurogenic inflammatory pain in rats which imply a possibility to use alternative therapies for pain control (Bowden et al 2017).

This study will explore how different types of chocolate can modify experimental pain. This by using intramuscular injections with hypertonic saline to induce pain and different types of chocolate, i.e. dark chocolate with content of 70% of cocoa, milk chocolate with a content of 34% of cocoa and white chocolate with a content of 30% of cocoa. The cocoa percentages and the type of chocolate are supposed to act as modifying factors of the characteristics of the pain (Martensson et al 2008).

To yield better evidence, there is a strive to keep the participant groups (men and women) as homogeneous as possible in every aspect.

**AIMS AND HYPOTHESIS**

The project aims to investigate if cocoa percentage affects the experimentally induced pain caused by hypertonic saline injection, in 15 healthy men and 15 healthy women. Investigation will also be made to whether chocolate preference can affect the response to the different types of cacao percentages.

**WORKPLAN**

*Hypotheses:* The hypothesis of the project is that the higher to cocoa content is the greater is the pain reducing effect. As a secondary hypothesis also the preference of the chocolate will have a significant pain reducing effect, but not as high as the content of cocoa.

*Design:* This study will include 15 healthy men and 15 healthy women with an age ranged from 18-40 years. The experiment will take place at the research lab at the Department of Dental Medicine, Karolinska Institutet, Huddinge, Sweden. The participants will attend at three sessions. Before the experiment, all participants will have baseline registrations of pain characteristics as well as pressure pain threshold which will be followed by an intramuscular injection of 0.2ml hypertonic saline (58.5 mg/mL active control substance) into the masseter muscle. The first five minutes after injection pain intensity (VAS 0-100) will be registered every 15^th^ second. After this pain characteristics and pressure pain threshold will be assessed every 5 minutes during 30 minutes and also map their pain distribution on pain drawings. After this, one piece of chocolate will be provided and 5 minutes later the injections of hypertonic saline will be repeated as well as the follow-up assessments.

At least one week of wash out will follow until the next session with another type of chocolate. During a single visit, one chocolate will be given to the participant containing either dark chocolate with a content of 70% of cocoa, milk chocolate with a content of 34% of cocoa and white chocolate with a content on 30% of cacao. The chocolates are all homogenous in color and shape and which makes them blinded. They will be given in a randomized and double-blinded order.

**MATERIALS AND METHODS**

The project comprises healthy subjects matched for age and gender which will be recruited among patients, volunteers, students and staff at Karolinska Institutet based on these factors:

*Inclusion criteria*: Age 18-40 year and pain-free.

*Exclusion criteria:* Systemic inflammatory connective tissue diseases, smokers, whiplash-associated disorder, fibromyalgia, neuropathic pain or neurological disorders, treatment with corticosteroids, obesity, diseases of salivary glands such as sialadenitis (e.g. Sjögren´s syndrome, viral or bacterial sialadenitis, obstructive sialadenitis) and salivary gland tumours, severe periodontal diseases and mucosal pain or ulcerations. Pain at the day of examination, e.g. headache, use of analgesics and other drugs and clinical examination/interview indicates a diagnosis of TMD according to the DC/TMD.

*Assessment of pain and emotional status:* A visual analogue scale (0-100) will be used for assessment of pain intensity. The pressure pain threshold (PPT) will be assessed with an electronic algometer. The patients will also map their pain distribution on pain drawings. In addition, validated questionnaires such as; PSS-10, GAD-7, PHQ-15, PHQ-9, PCS, OHIP, are used to assess psychological distress.

*Chocolate:* chocolate will be given to the participant containing either dark chocolate with a content of 70% of cocoa, milk chocolate with a content of 34% of cocoa and white chocolate with a content on 30% of cacao. The chocolates are all homogenous in color and shape and which makes them blinded. They will be given in a randomized and double-blinded order.

*Statistics:* According to the power calculation 12 volunteers (12 women and 12 men) are warranted for a power of 80 %, a significance level of 0.05, and to minimize the risk of losing power 15 will be included, however as a backup plan only 10 participants may be enough.

**CLINICAL SIGNIFICANCE**

The benefit of this study is increased knowledge and understanding of pain mechanisms and further how these can be affected. The hope is that this in turn can lead to improved diagnostic methods as well as more effective treatment of orofacial pain. This might in turn reduce the need for and costs of health care, and not the least, the patients’ individual pain and suffering.

**REFERENCES**

1. Goldberg DS, McGee SJ. Pain as a global public health priority. BMC Public Health. 2011;11:770.

2. Breivik H, Collett B, Ventafridda V, Cohen R, Gallacher D. Survey of chronic pain in Europe: prevalence, impact on daily life, and treatment. Eur J Pain. 2006;10(4):287-333.

3. Schiffman E, Ohrbach R, Truelove E, Look J, Anderson G, Goulet JP, et al. Diagnostic Criteria for Temporomandibular Disorders (DC/TMD) for Clinical and Research Applications: recommendations of the International RDC/TMD Consortium Network* and Orofacial Pain Special Interest Groupdagger. J Oral Facial Pain Headache. 2014;28(1):6-27.

4. Svensson P, Graven-Nielsen T. Craniofacial muscle pain: review of mechanisms and clinical manifestations. J Orofac Pain. 2001;15(2):117-45.

5. Louca Jounger S, Christidis N, Hedenberg-Magnusson B, List T, Svensson P, Schalling M, et al. Influence of Polymorphisms in the HTR3A and HTR3B Genes on Experimental Pain and the Effect of the 5-HT3 Antagonist Granisetron. PLoS One. 2016;11(12):e0168703.

6. Lovgren A, Haggman-Henrikson B, Visscher CM, Lobbezoo F, Marklund S, Wanman A. Temporomandibular pain and jaw dysfunction at different ages covering the lifespan--A population based study. Eur J Pain. 2016;20(4):532-40.

7. Pannucci CJ, Wilkins EG. Identifying and avoiding bias in research. Plast Reconstr Surg. 2010;126(2):619-25.

8. Bowden LN, Rohrs EL, Omoto K, Durham PL, Holliday LS, Morris AD, et al. Effects of cocoa-enriched diet on orofacial pain in a murine model. Orthod Craniofac Res. 2017;20 Suppl 1(Suppl 1):157-61.

9. Martensson L, Stener-Victorin E, Wallin G. Acupuncture versus subcutaneous injections of sterile water as treatment for labour pain. Acta Obstet Gynecol Scand. 2008;87(2):171-7.

10. Dao, TT., LeResche, L., Gender differences in pain, J Orofac Pain. 2000;12(3):169-84

11. Hallberg, R.-M., Carlsson, S. G. Coping with Fibromyalgia 2013;1(14):29-36

12. Thomas, M. E., Weiss, S. M. Nonpharmacological Interventions with Chronic Cancer Pain in Adults. 2000;(10).

13. Yokoyama, T., Maeda, Y., Audette, K. M., Sluka, K, A. Pregabalin Reduces Muscle and Cutaneous Hyperalgesia in Two Models of Chronic Muscle Pain in Rats. 2007;(8):422-429.

14. Lesurtel, M., Soll, C., Graf, R., Clavien, P-A. Role of serotonin in the hepato-gastronIntestinal tract: an old molecule for new perspectives. 2008;(65)940-952.

15. Silva NR. Chocolate consumption and effects on serotonin synthesis. Archives of internal medicine. 170(17):1608; author reply 1608–1608; author reply 1609

16. Nehlig, A. The neuroprotective effects of cocoa flavanol and its influence on cognetive performance. 2013; 75(3): 716-726.
