# Supplementary material for: Chocolate intake and muscle pain sensation: A randomized experimental study
Source: PLoS One. 2023 May 24;18(5):e0284769. doi: 10.1371/journal.pone.0284769 (PMC10208501; doi:10.1371/journal.pone.0284769)
Supplement: Attachment — Submitted filename: R1-Response to reviewers.docx [file pone.0284769.s004.docx]

**Response to reviewers:**

**PONE-D-22-13220**

**Cocoa intake and muscle pain sensation: a randomized experimental study**

**Nikolaos Christidis, 2022-12-04**

Dear Academic Editor,

Thank you for considering this manuscript for publication. Since two of the reviewers approved the statistics and the third had some concerns, we have tried to address some parts of the statistics. The methods and set-up for this study have been used in several (hundreds) publications with hypertonic saline as experimental pain instigator, so any carry over effects have been taken into consideration in the study plan, thus not affecting the results. Furthermore, we have several publications regarding this model using the same statistics after consultation with a biomedical statistician, and this statistical method has also been approved in three doctoral theses from our group alone (Christidis Nikolaos 2010, Sofia Louca Jounger 2017, and Samaa Al Sayegh 2021). However, as it was suggested to use one statistical method for all analyses, we discussed using 2-way repeated measures for all analyses. Since data was not normally distributed we chose the more conservative non-parametric statistical Friedman repeated measures ANOVA method. In fact, we have already presented the pre-and post-chocolate effect difference for the analysis; we have also used the peak pain value for analysis, but since it was not obvious for the reviewer we have clarified that section. Finally, we do not have the statistical programs suggested by the reviewer, so we have to use SigmaPlot. Therefore, we hope that you still consider this manuscript based on the fact that the method used has been approved several times before.

We would like to thank all three reviewers for the comments and suggested changes in order to improve the quality of this manuscript. The changes are highlighted in yellow. We hope that this reply and changes in the manuscript are sufficient and will answer your queries.

**Reviewer 1:**

***** General comments: *****

The design is a three-period three-treatment crossover design with the further complication of including a two-factor within-subject design with multiple evaluations over time in each period. This design requires special techniques to analyze correctly. See Senn (2002) for details about methodology. The current analysis is incorrect.

The manuscript lacks line numbers, therefore, it is difficult to provide specific suggestions. There are minor grammatic errors that might benefit from a third-party proof-reading.

In addition, it does not seem that the data are available for review.

***** Specific comments: *****

The authors presumably have already found that their design is not going to be handled correctly using SigmaPlot -- however, the repeated measures analysis that is proposed is incorrect. Also, it seems likely that the implied R-side correlation structure implemented by SigmaPlot is too simplistic. Further, the use of the Friedman test is even more limited in its ability to correctly analyze these data.

This crossover design features:

* 3 periods

* 3 treatments

* 6 sequences

* 30 subjects

The within period design features:

* 2 conditions (pre- and post-chocolate)

* Multiple time-points

* Multiple measures

While it is not clear that performing a correct analysis would lead to substantially different results, it is difficult to say ahead of actually performing it. A correct analysis will include treatment, sequence, subject, period, and carryover effects in the between-subject design, and pre/post and time in the within-subject design. It may be reasonable to omit the carryover, but this must be justified. Senn (2002) provides an excellent discussion of this issue that may be of help.

As mentioned in the limitations, there is most likely a period effect. Therefore, this must be accounted in the analysis at a minimum. The randomization restriction represented by the sequence is also important. A subject-level effect is required to produce the correct degrees of freedom for analysis (and potentially, depending on how the analysis is specified, a random effect of subject by period). Most likely, the authors would like to generalize their results past this specific set of subjects, so that subject should be considered as a random effect.

This design should probably be analyzed using either SAS or R. It is recommended to use SAS, unless the authors have access to expertise in using R to specify complex analyses. In SAS it would be feasible to implement the entire design as run using PROC MIXED. However, setting up the analysis does require a little experience, effort, and likely some trial and error and it would require both G-side and R-side specifications.

Having set this analysis up would provide several benefits. First, it allows all tests of interests to be performed as either omnibus tests or contrasts (whether corrected for multiple testing or not). Second, it allows covariates to be entered to evaluate their effects net of the treatment or vice versa. Third, it provides a template that correctly reflects the experiment design in all its complexity and can be used for all assessments (ideally).

Alternatively, while the fine-grained sampling over time is interesting, it seems to provide little benefit in the actual analysis. Therefore, a simpler method of analyzing the data would be as follows:

* Summarize the data across time using a statistic or statistics, e.g.:

- Area under the curve

- Peak value

- Other

* Determine the differential effect of chocolate using the summary statistic, e.g.:

- Post-chocolate minus pre-chocolate

- Ratio of post-chocolate to pre-chocolate

* Perform a standard 3-treatment 3-period crossover analysis

Some sample PROC MIXED code for this "simpler" analysis would be:

PROC MIXED;

CLASS SEQUENCE SUBJECT PERIOD TREATMENT;

MODEL DELTA = TREATMENT PERIOD;

RANDOM SUBJECT(SEQUENCE);

LSMEANS TREATMENT / PDIFF CL E;

RUN;

(Note that the code above could be extended to allow the pre/post x time design within subjects, and this would indeed be the starting point for that effort.)

A side benefit of this approach is that it allows an easier comparison of the various measures with each other via scatterplots or correlation analysis. It is also more likely to provide data that meet the assumptions of the linear model analysis.

The authors state that they evaluate normality of the data. This is a misnomer. One can easily see that in any case of interest with actual group differences that the data will not be normally distributed. What is assessed is the normality of the residuals from the linear model analysis. (Or, as in this case, suitably standardized or studentized residuals.)

Even if these residuals are not completely consistent with normality, it is usually preferable to use the same analysis structure throughout rather than a hodge-podge of statistical methods. Nonparametric analogues can be used as support for this. Recall that linear models still produce unbiased results even if error distributions are not normal. This pays dividends in interpretability and ease of representing the experimental design.

Note that the authors present significance tests based on ranks, but then quantitative results based on means. This is a common method to try to dodge the fact that rank-based methods provide no real quantitative statements but strikes one as disingenuous. Using a linear model approach is better for this purpose, even if the model fit is only fair.

***** Reference: *****

Senn, S. S. (2002). Cross-over trials in clinical research (Vol. 5). John Wiley & Sons.

**Reply:**

**Thank you for reviewing this manuscript. We have added line-numbers in the version with the changes tracked.**

**When it comes to the concerns it seems that your major concern is the statistical method used. We have also been told by our biostatisticians to use non-parametric statistical methods for not normally distributed data as well as categorical and ranked data, even though they are more conservative. That is why we have used the Friedman repeated measures test for peak pain, pain duration and pain area.**

**Some of your concerns regard a carry-over effects. In this case each subject is its own control so we have used the pre- and post-chocolate difference as you suggested. However, since this was not obvious, we have clarified it in the intake of chocolate session as well as in the statistics section.**

**You also suggested that we should use simpler statistics such as the peak pain intensity difference, which already is presented in the results under its own section.**

**Further, there are thousands of publications on hypertonic saline as experimental setting and our group has used this method in several studies and in three theses that have been approved.**

**A final reason for not using more tests is that we do not wish to “significance-fish”.**

**Unfortunately, we do not have the statistical programs suggested, nor the knowledge to use the R system. Therefore, we use the analysis previously used and approved as suggested by our statisticians. We hope this response, where we consider your suggestion to use one method instead of mixing, where we have the pre- and post-chocolate difference, and the clarification in the text is acceptable for you.**

**Reviewer 2:**

This paper reports on a generally well-designed study producing novel data that intake of chocolate reduces acute pain induced by hypertonic saline injection into the masseter muscle of young healthy men and women. There are however several points that need to be satisfactorily addressed by the authors before it could be considered acceptable.

1. The first 4 paragraphs of the Introduction inexplicably focus on chronic pain, yet this study did not address chronic pain per se since it used an experimental model of acute pain which the Discussion states is used “to mimic clinical acute muscle pain”. The 1st part of the Introduction thus needs to be re-cast with a focus on acute pain and underlying mechanisms, including those expressed in the masticatory muscles.

**Reply: Thank you for this comment. The first part of the introduction has changed focus to acute, experimental pain as suggested.**

2. The introduction’ s middle paragraph about cocoa inhibiting “trigeminal nerve cells……spinal cord” needs modification since trigeminal nerve cells predominantly project to the brainstem, not spinal cord.

**Reply: This has been corrected accordingly.**

3. There seems to be a disconnect in the description of the Study Design. Clearer wording is needed. Items in pain scores are said to have been graded on a 0-10 scale but this is followed by the statement that they were graded from 0 to 4.

**Reply: This part has been re-written to clarify any concerns.**

4. Timelines are given in the Methods text and Flow Chart but It is not convincing that there was sufficient time for chocolate ingestion to have occurred to exert analgesic effects (eg, via modulation of neurogenic inflammation) especially during the initial time points where the pain reductions are extremely modest. Also, the time designation on the Time axis in Figs 3 and 4 are tiny and very difficult for the reader with even 20/20 vision to discern.

**Reply: You are right; however, since this was the first study of its kind we did not know and chose to use the standard time-points for this experimental pain model. Perhaps time was not sufficient, but that requires a new study with different timelines. This is addressed in study limitations.**

**The figures might have been downsized in the system for the review. They have a high resolution of 900 dpi and can be zoomed in easily.**

5. The Study Design outlines measures to assess both pain intensity and pain disability, but the Results overlooks any presentation of the latter.

**Reply: This was misleading in the study design; no such assessment or analysis was performed since the study aimed at pain characteristics. This has been clarified.**

6. Strengths of the study included the use of different concentration of cocoa in the chocolate pieces ingested, and the finding that taste preference played a key role. The 1st couple of paragraphs of the Discussion discuss cocoa and its analgesic effects but it is unclear how a definitive conclusion could be reached from the present study that it is the cocoa in the ingested pieces of chocolate that was responsible for the modest analgesic effects. There were other ingredients in the chocolate (soy, sugar, vanilla) and there is no indication in the study design that these were controlled for, despite different cocoa concentration being used. Accordingly, the title of the paper should be modified to reflect “chocolate” not ‘cocoa’.

**Reply: The title has been changed accordingly**

7. The 1st sentence of the Discussion does not make sense; it seems to be missing words.

**Reply: This has been adjusted accordingly**

8. Likewise, the 1st sentence of the 9th paragraph about taste preference being “responsible for the response to cold pain tolerance” is unclear and needs to be re-formulated to make it clear how taste preference could affect a “response” to the “tolerance” of “cold pain”. The next 2 sentences also have a problem in the formatting (and show grammatical errors).

**Reply: This part has been adjusted as suggested.**

9. The Study Limitations will need to include those identified above (eg, Point 4).

**Reply: This has been added as you pointed out.**

**Reviewer 3:**

The subject could be of great interest however, the design and the interpretation of the results are a limitation.

The main concern is that the authors do not know or not show the composition of the chocolate. White chocolate has only cocoa fat with no flavanols at all (30% of cocoa fat is a lot really). They omit the sugar content in the different chocolates that should be around 50% for milk and white chocolate. Maybe these facts can better explain the preference and the un-expected results regarding the "higher flavanols the higher analgesic effect". I think that, unfortunately, it is not possible to get to any conclusion because there is no real control for the effect. It looks to me that it is the sugar content the one producing any effect and to probe it you would need a non -sugar content control

**Reply: We have added the composition of the chocolates and clarified in the first paragraph of the discussion that there are other, more probable factors that can explain the positive outcome, as suggested. This was also addressed in the study limitations and conclusion (as suggested further down).**

When authors say that is a controlled study: Which is the control?

**Reply: Each participant is its own control. First, they get an injection with hypertonic saline, then after half an hour chocolate, and 2 minutes later a new injection with hypertonic saline. Therefore, the control is pre-chocolate injection vs post-chocolate injection in each subject and the analysis is based on that. This has been clarified according to your question.**

The conclusion “The results indicate that it is not mainly the cocoa-concentration that has a positive effect on pain-characteristics, but the effect seems to be affected by the preference” is not based in the results. There is no significance or correlation to support it. Again I think the difference lays in the sugar content.

**Reply: This has been adjusted as mentioned above.**

I don’t see the point of using an abbreviation for serotonin.

**Reply: This has been changed, as suggested.**

Did the authors check for a sequence effect in the assays (white, milk or dark) on the preference for one or other chocolate?

**Reply: Unfortunately, that data does not exist for one of the sexes, just which one they preferred. That is why we presented it in the “Demographic data”.**
