# Supplementary material for: Chocolate intake and muscle pain sensation: A randomized experimental study
Source: PLoS One. 2023 May 24;18(5):e0284769. doi: 10.1371/journal.pone.0284769 (PMC10208501; doi:10.1371/journal.pone.0284769)
Supplement: Attachment — Submitted filename: R2-Response to reviewers.docx [file pone.0284769.s005.docx]

**Response to reviewers:**

PONE-D-22-13220 R1

Cocoa intake and muscle pain sensation: a randomized experimental study

Thank you for further comments and suggestions to our manuscript.

Since some questions about the statistical analysis remain, we have tried to address them in the revised text.

Please note that by reviewing the entire manuscript, some of your suggested changes (with specific lines) might have shifted. We also took the opportunity to tie up the manuscript further.

Our point-by-point answer to your queries are presented in **bold** *italics*, ahead.

The changes are highlighted using the track changes function in Word. We hope that these changes answer too all your queries.

**Review Comments to the Author**

Please use the space provided to explain your answers to the questions above. You may also include additional comments for the author, including concerns about dual publication, research ethics, or publication ethics. (Please upload your review as an attachment if it exceeds 20,000 characters).

**Reviewer #2**:

The authors have satisfactorily addressed my remaining points from the previous review, except for point 7 since the first sentence still is unclear: how could it be that findings that “the pain reduction by the different types of chocolate presented in this study is line with previous studies “ (11, 48-50); none of these 4 cited studies investigated the effects of chocolate on pain. The sentence seems unnecessary and could be deleted unless the authors modify this sentence to clarify exactly what they mean by this unclear sentence.
***R: Thank you for your comment, and sorry for the confusion. We were referring to the studies that used the same procedure (e.g., pain-induced in the masseter), not chocolate. We have edited that sentence accordingly.***

Also note the revised wording on line 211 has introduced inappropriate tense changes in the same sentence; past tense should be used for both sets of verbs.

***R: We are sorry for that oversight. We have corrected that sentence.***

**Reviewer #3**:

I think that the composition of the chocolates, now that it has been included, clearly shows that the effect, if any, is not due to the bioactives in cocoa, polyphenols and methylxanthines, but to maybe the cocoa fat and the sugar. In fact, the results show that in the case of men the sugar and fat content with no cocoa bioactives is more effective than the bioactive containing cocoa of dark chocolate.
I think that all this should be included in the abstract. It does not seem to be a question of preference but a question of sugar (and maybe fat contents)
This is in disagreement with most papers showing an effect of cocoa polyphenols and methylxanthines at different levels
See for instance:
Goya L, Kongor JE, de Pascual-Teresa S. From Cocoa to Chocolate: Effect of Processing on Flavanols and Methylxanthines and Their Mechanisms of Action. Int J Mol Sci. 2022;23(22):14365. Published 2022 Nov 18. doi:10.3390/ijms23221436
or
Gu Y, Lambert JD. Modulation of metabolic syndrome-related inflammation by cocoa. Mol Nutr Food Res. 2013;57(6):948-961. doi:10.1002/mnfr.201200837
etc
However, I think the paper should be published showing the results they got with really low amounts of cocoa solids (0% in white chocolate and only 9% in milk chocolate).
It is very important to show that this assay was done based on the bioactivity of cocoa (polyphenols and methylxanthines) however, the real content of this compounds, that was not determined, should be really low since cocoa fat has not beneficial effects that we knew until now.

***R: Thank you for your suggestion and support to our findings. We have now reworded the findings in both the abstract and the text to highlight that it was the sugar content (and low amounts of cocoa solids) rather than the cocoa content alone that influenced the pain sensation.***

**Reviewer #4**: The paper presents a randomized double-blinded controlled trial. First and foremost, the most important question if this is a powered trial to detect a significant effect. With 30 samples and without any power analysis this is not a powered trial. So any claim of significance based on p-value does not make sense.
***R: Thank you for your comment, appreciated. We performed power calculation with our statistician to confirm that we had a strong power (>0.999) to detected any difference, significantly.***

Second, I reviewed the previous round of reviews and agree with the statistical reviewer that this should be analyzed using a crossover design. A mixed-effect model with a period effect should be used. The previous reviewer has given some excellent guidelines on how to analyze the data which I agree with. He/she also has SAS code, which was pretty tough. Unfortunately, all the suggestions are ignored for two reasoning. 1. They do not have access to SAS and 2. The authors have used similar simpler methods earlier which are accepted.
This is not a constructive way to address the reviewer’s suggestions. First, if SAS is not available, please use R, which is completely free to use. Second, just because previous papers are not reviewed by a statistician thoroughly it does not mean the methods used by them are all appropriate. It seems the investigative teams lack a statistician as otherwise converting the given SAS code to R and reanalyzing the data using a crossover design is not a big issue. The reviewer all mentions several other challenges that have to do with normality and model fitting which I cannot comment on given the suggestion is ignored.

***R: We do welcome both reviewers’ comments and suggestions. We are sorry if we came across as dismissive of your concerns, that was not the intention. We were trying to explain that SPSS did the work that was aimed to be done in our study, and we (and others) have used before. We know it is not the only way to perform statistical analysis while recognising that SPSS (point-click based) has been used in trials of the same design by many other researchers – yet, it does not make it infallible compared to SAS (programme based). We consulted with out statistician to confirm our analysis and we have also added the discussion around limitation to address this issue. We also revised the entire manuscript to better adequate the language used to present and discuss the results in light of the potential limitations. Thank you very much for understanding.***
