# Supplementary material for: Chocolate intake and muscle pain sensation: A randomized experimental study
Source: PLoS One. 2023 May 24;18(5):e0284769. doi: 10.1371/journal.pone.0284769 (PMC10208501; doi:10.1371/journal.pone.0284769)
Supplement: Attachment — Submitted filename: R3-Response to reviewers final.docx [file pone.0284769.s006.docx]

**Response to reviewers:**

PONE-D-22-13220 R2

Chocolate intake and muscle pain sensation: a randomized experimental study

Thank you for further comments and suggestions to improve our manuscript, in this case the abstract.

Our point-by-point answer to your queries are presented in **bold** *italics*, ahead.

The changes are highlighted in yellow. We hope that this reply and changes in the manuscript are sufficient and will answer your queries.

**Review Comments to the Author**

Please use the space provided to explain your answers to the questions above. You may also include additional comments for the author, including concerns about dual publication, research ethics, or publication ethics. (Please upload your review as an attachment if it exceeds 20,000 characters).

**Reviewer #2**:

The authors have satisfactorily addressed the remaining points in my most recent review. However, I do not believe that the authors have satisfactorily addressed the point of Reviewer #3 (which was also related to a point that I raised in an earlier review) that sugar or other ingredients in the chocolate could have contributed to the reduction in pain and that this should be included in the Abstract. The revised Abstract now in its conclusion only refers to taste preference for sugar to explain the pain reduction, even though now the Discussion (lines 684-685) indicates that “other ingredients in the chocolate that could explain this chocolate-induced reduction of pain variables” and the Conclusion (lines 708-709) states that “Another possible explanation could be the composition of the chocolate, i.e. the concentration of the other ingredients such as sugar, soy, and vanilla. Comparable wording should be briefly reflected in the Abstract conclusion.
***R: Thank you for your comment, and sorry for the confusion, that was not our intention. We have adjusted the conclusion making the wording comparable to the discussion and conclusion in the manuscript, as suggested.***

**Reviewer #3**:

The abstract keeps being unclear and now I think that intentionally misleading. White chocolate might have 30% cocoa fat, but no flavanols at all (introduction and hypothesis is based on this). The results clearly show that if any the pleasure of fat plus sugar intake (that is what white chocolate and milk chocolate mainly provide) is the one producing the "analgesic" effect, very interestingly and in a statistically significant way in the case of white chocolate in men. I think that this needs to be said from the abstract.

***R: Thank you for your comment, and sorry for the confusion, that was not our intention. We have adjusted the conclusion making the wording comparable to the discussion and conclusion in the manuscript, as suggested.***

**Reviewer #5**: The authors, in this round of revision, did their best to address the concerns raised. However, they relegated the suggestion of conducting a full-scale crossover design. It is not clear why they were reluctant to conduct it. I am OK to pass the paper, but more arguments are needed behind not using a cross-over design as a separate analysis.
***R: Thank you for your observation. We now expanded a bit further on the reasoning of not utilizing a full-blown design. We did use some of its premises, including patients being their own control, and sing a smaller sample size. However, the duration of the study might have hindered a full-scale design. We have added this point into the limitations.***
